# Posttraumatic stress disorder in early childhood: classification and diagnostic issues

**DOI:** 10.3402/ejpt.v4i0.21357

**Published:** 2013-12-20

**Authors:** Alessandra Simonelli

**Affiliations:** Department of Developmental and Social Psychology, Padova University, Padua, Italy

**Keywords:** posttraumatic stress disorder, early childhood, 0–3 diagnostic classification, PTSD alternative algorithm, developmental trauma disorder

## Abstract

The 0–3 diagnostic classification of infant mental health, on the basis of DSM-IV-R, describes posttraumatic stress disorder (PTSD) as a pattern of symptoms that may be shown by children who have experienced a single traumatic event, a series of connected traumatic events, or chronic, enduring stress situations. This definition, related to young children, needs the consideration of several factors to understand the child's symptoms, organize the diagnostic process, and realize clinical interventions. In this sense, the clinician must appreciate the classification criteria of PTSD in early childhood in the context of the child's age, temperament, and developmental level. This report presents a review of the research in the domain of the PTSD in early childhood with particular attention to the developmental considerations to define critical diagnostic criteria, specifically organized on the child characteristics, competences, and needs. Along this line, it will describe two proposed modifications of the diagnostic classification in childhood: the Post Traumatic Stress Disorder Alternative Algorithm (PTSD-AA) and the definition of developmental trauma disorder (DTD).

According to the review studies by Scheeringa, Zeanah, and Cohen (2011) and Koenen, Roberts, Stone, and Dunn (2010) on community samples of either adults or children (both in infancy and in early childhood), 40–68% of the subjects had experienced at least one potentially traumatic event and 37% had been exposed to more than one. It has been well established from studies on adult populations that while most individuals are resilient to trauma exposure, roughly 30% of traumatized adults will develop posttraumatic stress disorder (PTSD) (Kessler, Sonnega, Bromet, Hughes, & Nelson, 1995) and of these subjects, approximately 50%, will have an unremitting and impaired course of consequences (Davidson & Fairbank, 1993).

Similar to adults, data on children clearly show that post-traumatic symptoms commonly occur following exposure to a traumatic event and don't quickly remit in the course of development.

The studies reveal that 75% of children with PTSD also had at least one comorbid disorder and over 50% of these first occurred after their traumatic experiences (Scheeringa & Zeanah, 2008). The most commonly associated conditions are: oppositional defiant disorder (ODD, 75–61%), separation anxiety disorder (SAD, 68–21%), attention-deficit/hyperactivity disorder (ADHD, 38–33%), and major depressive disorder (MDD, 43–6%) (Scheeringa & Zeanah, 2008; Scheeringa, Zeanah, Myers, & Putnam, 2003). These results highlight that over half of these comorbid disorders started after the traumatic experiences; furthermore, none of the new-onset comorbid disorders existed earlier and in the absence of PTSD symptoms. In other words, non-PTSD disorders that arose following traumatic experiences were always accompanied by PTSD symptomatology (Sheeringa, 2009).

In addition, considering the course and prognosis of the PTSD in infancy and early childhood, the study by Scheeringa, Zeanah, Myers, and Putnam (2005) observed 35 children (who were 1–6 years old during the first assessment) for 2 years: the authors revealed no statistically significant decrease in the number of PTSD items over time. These results are consistent with research by: Laor, Wolmer, Mayes, and Gershon (1997) on preschool children exposed to Scud missile attacks in Israeli, McFarlane (1987) on school age children exposed to an Australian bush fire, Shaw (1996) on children exposed to Hurricane Andrew and Meiser-Stedman, Smith, Glucksman, Yule, & Dalgleish (2008) with a study on motor vehicle accidents. In all of these cases, the PTSD symptoms showed no significant change or decrease in the time after traumatic experiences until 18–21 months after the trauma.

These data, despite limits regarding methodological issues such as data selection, collection and analysis (Koenen et al., 2010), highlight that: (1) infants and children are exposed to a high number of traumatic (or potentially traumatic) experiences; (2) these experiences elicit post-traumatic symptoms that do not seem to remit spontaneously; (3) these symptoms require an accurate diagnosis, which needs appropriate developmental diagnostic criteria; (4) the PTSD in infancy and early childhood is a central clinical phenomenon that requires adequate treatment on the basis of a development oriented diagnosis.

The greatest challenge in the study of PTSD in childhood, specifically in early childhood, is represented by the changing presentation of (essentially the same) symptoms in the course of the child's development as well as changes in the nature of symptoms. For example, difficulties in self-regulation change significantly with developmental phases and the individual child's competencies: loss of bladder control in a 6-year-old child can be a sign of traumatic dysregulation, while it is not for a 2-year-old child. In other words, these modified definitions of early childhood PTSD symptoms must consider the speed of the child's development and changes, especially during the first year of life (see Section 4). These transformations certainly modify the meaning of the symptom, but also its nature, creating a need for appropriate and sensible models to identify these modifications, acting as a guide for a specific diagnosis directed by the (affective-relational and cognitive-behavioral) developmental approaches.

## 1. Classification of posttraumatic stress disorder in early childhood

The main interest in the classification and diagnosis of PTSD in early childhood requires focus on the following aspects: (1) the definition of “trauma” and “traumatic events” considering child's age and development, familiar and social conditions; (2) the definition of specific displays of the disorder in children (van der Kolk & d'Andrea, 2010); and (3) the role of subjective components of the reaction to the traumatic event.

According to the DSM-IV classification (American Psychiatric Association, 1994), PTSD is connected to a preliminary condition: the subject's exposure to a traumatic event (Criterion A); the definition of trauma describes situations in which “the person experienced, witnessed, or was confronted with an event or events that involve actual or threatened death or serious injury; or a threat to the physical integrity of himself or herself or others” (American Psychiatric Association, 1994, p. 427). The stressor definition also includes a subjective component which requires the individual to respond to the trauma with “intense, fear, helplessness, or horror” (American Psychiatric Association, 1994, p. 428). In other words, the subjective component acknowledges that the personal reaction to a trauma plays a crucial role in determining the development of PTSD (Salmon & Bryant, 2002).

The primary symptoms of PTSD are divided into three clusters (Table 1).

**Table 1:** DSM-IV criteria for posttraumatic stress disorder (PTSD) (American Psychiatric Association, 2000b)

A: Person was exposed to a traumatic event in which both were present:
(1) person experienced, witnessed or was confronted with event(s) involving actual or threatened death, serious injury, or threat to physical integrity of self or others;
(2) person's response involved intense fear, helplessness or horror; in children may be expressed by disorganized or agitated behavior.
B: Traumatic event is persistently re-experienced in at least one of the following:
(1) recurrent and intrusive distressing recollections of the event including images, thought or perceptions; in young children repetitive play in which trauma themes are expressed;
(2) recurrent distressing dreams of the event; in children frightening dreams with no recognizable content;
(3) acting or feeling as if the traumatic event were recurring, reliving illusions, hallucinations, dissociative flashbacks; in young children trauma-specific re-enactment;
(4) intense psychological distress at exposure to reminders of the traumatic event;
(5) intense physiological distress at exposure to reminders of the traumatic event.
C: Persistent avoidance of trauma reminders and new numbing of general responsiveness, indicated by at least three of the following:
(1) efforts to avoid thoughts, feelings, or conversations about the trauma;
(2) efforts to avoid activities, places, or people that arouse memories of the trauma;
(3) inability to recall an important aspect of the trauma;
(4) markedly diminished interest or participation in significant activities;
(5) feeling of detachment or estrangement from others;
(6) restricted range of affect;
(7) sense of a foreshortened future.
D: Persistent new symptoms of increased arousal as indicated by at least two of the following:
(1) difficulty falling or staying asleep;
(2) irritability or angry outbursts;
(3) difficulty concentrating;
(4) hypervigilance;
(5) exaggerated startle response.

The first cluster (Criterion B) involves re-experiencing symptoms, like intrusive memories, nightmares, a sense of reliving the trauma, or psychological or physiological distress when remembering the traumatic situation. This criterion is specified for children by these notes “in younger children, distressing dreams of the event may, within several weeks, change into generalized nightmares of monsters, of rescuing others, or of threats to self or others” or “there may be frightening dreams without recognizable content” (American Psychiatric Association, 1994, p. 428). Furthermore, it is proposed that “young children usually do not have a sense that they are reliving the past; rather, the reliving of the trauma may occur through repetitive play (e.g., a child who was involved in a serious automobile accident repeatedly reenacts car crashes with toy cars)” (American Psychiatric Association, 1994, p. 428).

The second cluster of symptoms refers to avoidance patterns (Criterion C) and, to get closer to the criteria, the person needs to feel at least three of the following symptoms: persistent avoidance of thoughts, feelings, and reminders of the trauma; inability to recall some aspects of the trauma; withdrawal from others and normal activities; emotional numbing and a sense of a foreshortened future. It is acknowledged that the assessment of these features in childhood PTSD may be difficult because of children's potential difficulties in reporting subjective reactions (Salmon & Bryant, 2002). Specifically, it is stated that “because it may be difficult for children to report diminished interest in significant activities and constriction of affect, these symptoms should be carefully evaluated with reports from parents, teachers, and other observers” (American Psychiatric Association, 1994, p. 426). This aspect contemplates the fact that children's ability to monitor their psychosocial functioning depends on their developmental level, a consideration that needs to be accommodated in the assessment of PTSD.

The final cluster involves arousal symptoms (Criterion C), and the person needs to experience at least two of the following symptoms: insomnia, irritability, difficulty in concentration, hypervigilance, or heightened startle response. The specifications for children note that the child “may also exhibit various physical symptoms, such as stomachaches and headaches” (American Psychiatric Association, 1994, p. 426).

In line with the DSM-IV-R (American Psychiatric Association, 2000b), the 0–3 diagnostic classification of infant mental health (Zero to Three, 2005) also describes the PTSD as a pattern of symptoms that may be shown by children who have experienced a single traumatic event, a series of connected traumatic events, or chronic and enduring stressful situations. In this classification system, this type of clinical disorders in childhood is included on Axis I. As described, the trauma may be a sudden and unexpected event (e.g., car accident, shooting), a series of connecting events (e.g., repeated air raids), or/and enduring situation (e.g., chronic child battering, sexual abuse). A fundamental criterion for the diagnosis is the interference of the symptoms with daily functioning and their duration for at least 1 month. Symptoms must be also understood in the context of the trauma, the child temperament, personality characteristics, and developmental level. From this point of view, the diagnosis of PTSD requires that all five of the following criteria must be met (Zero to Three, 2005, pp. 16–17, Table 2):

**Table 2:** 0–3 Classification criteria for posttraumatic stress disorder (PTSD) in early childhood (0–3, Zero to Three, 2005)

(1) The child has been exposed to a traumatic event that threat to the physical or psychological integrity of the child or another person
(2) The child shows evidence of re-experiencing the traumatic event(s) by at least one of the following symptoms:
(a) Posttraumatic play that represent a reenactment of some aspects of the trauma, is compulsively driven, fails to relive anxiety and is more literal and less elaborate and imaginative than usual.
(b) Recurrent and intrusive recollections of the traumatic event outside play.
(c) Repeated nightmares the content of which may or may not be linked to the traumatic event.
(d) Physiological distress expressed in language or behavior.
(e) Recurrent episodes of flashback or dissociation.
(3) The child experiences a numbing of responsiveness or interference with developmental momentum by at least one of the following symptoms:
(a) Increased social withdrawal;
(b) Restricted range of affects;
(c) Diminished interest or participation in significant activities;
(d) Efforts to avoid activities, places or people that arouse recollection of the trauma, including thoughts, feelings, and conversations associated with the trauma.
(4) A child may exhibit at least two of the following symptoms of increased arousal, after traumatic event:
(a) Sleep difficulty;
(b) Difficulty concentrating;
(c) Hypervigilance;
(d) Exaggerate startle response;
(e) Increased irritability, anger or extreme fussiness, or temper tantrums;
(5) Persistence of this pattern of symptoms for at least 1 month.

As discussed, these two definitions and classification criteria (DSM-IV and 0–3 classification) determine a fundamental issue regarding the validity of PTSD diagnosis in early childhood. As a matter of fact, studies that have systematically examined the diagnostic criteria for PTSD in preschool years attest that the disorder can be reliably detected in young children that manifest most (but not all) of the items (Sheeringa, 2009). In other words, the DSM-IV-TR posttraumatic stress disorder criteria do not adequately capture the symptom manifestations experienced by children, particularly infants and preschoolers, and underestimate the number of children experiencing considerable post-traumatic distress and impairment (Carrion, Weems, Ray, & Reiss, 2002; Iselin, Le Brocque, Kenardy, Anderson, & McKinlay, 2010; Meiser-Stedman et al., 2008; Scheeringa, Zeanah, Drell, & Larrieu, 1995).

The limitations of DSM-IV criteria in the diagnosis of PTSD in childhood might serve as a basis to present a central question is this domain, that is, whether there is any evidence that PTSD exists in early childhood. As argued, undoubtedly children at this age experience a large number of serious and potentially traumatic events but, despite this exposure, several studies examining frequencies of PTSD using DSM-IV criteria have found them to be surprisingly low. In samples of traumatized young children in which we might expect a high prevalence of PTSD, the frequencies of PTSD diagnosis ranged only from 0 to 20% (cf. Sheeringa, 2009), whereas a community study of 2–5-year olds found a prevalence rate of 0.1% (Egger et al., 2006).

There are several possible explanations for these data: first, the fact that young children are relatively protected, perhaps by their cognitive and affective immaturity, from developing PTSD following the experience of trauma. This is a sort of “resilience-based” point of view that considers an auto-protective mechanism capable of preserving young children from PSTD symptoms, in response to the trauma exposition. However, it is possible that the low prevalence of PTSD in early childhood results from of a lack of sensitivity of the diagnostic criteria that are not developmentally appropriate to detect the presence of manifestations of the disorder at this age.

Fletcher's (1996) meta-analysis on this theme indicated that the incidence of specific PTSD symptoms in children in preschool years and adults are comparable, including intrusive memories (34% and 45% for children and adults, respectively), nightmares (31% and 36%), reliving the event (39% and 29%), distress in response to reminders (51% and 26%), avoidance of reminders (32% and 33%), diminished interest in activities (36% and 28%), concentration difficulties (41% and 41%), hypervigilance (25% and 27%), and exaggerated startle response (28% and 38%). Fletcher (1996) reported other problems in traumatized children, including dissociative responses (48%), low self-esteem (34%), depression (25%), separation anxiety (23%), and generalized anxiety (39%). Similarly, confirmatory factor analyses of PTSD reactions of 5,664 children and adolescent victims of Hurricane Hugo have found three symptom clusters responses: intrusion/active avoidance, numbing/passive avoidance, and arousal (Anthony, Lonigan, & Hecht, 1999), which are comparable to symptom clusters observed in adult populations (Taylor, Kuch, Koch, Crockett, & Passey, 1998). Nevertheless, comparability between children's and adults’ reactions should not be interpreted as evidence that childhood and adulthood PTSD are identical conditions: for example, many studies attest that preschool children show more circumscribed symptoms than older children, as fewer cognitive symptoms (e.g., minimal re-experiencing symptoms) and little avoidance (e.g., inability to recall an aspect of the trauma and avoidance of thoughts, feelings, or conversations about the event). In general, McNally (1993) notes that the variability of findings concerning childhood PTSD might be expected due to uncertainty about the extent to which children have been asked about specific symptoms and the reliability of assessment tools that have been used to index symptoms.

This second point of view constitutes, therefore, the main problem in the PSTD diagnosis by the DSM-IV classification and the reason is because a number of alternative diagnostic algorithms and modifications of the existing criteria have been proposed to take into account how trauma reactions typically present in childhood. Of particular note are the alternative PTSD algorithm (PTSD-AA; Scheeringa et al., 1995) and the developmental trauma disorder (DTD; van der Kolk, 2005a) described to consider the complex trauma in childhood.

## 2. An alternative PTSD algorithm

Scheeringa et al. (2003) suggest to take into account an alternative criteria algorithm (PTSD-AA; Scheeringa et al., 1995) that includes modifications in wording for several items to make them more developmentally sensitive to child populations. The PTSD-AA was developed by modifying DSM-IV-TR PTSD symptom wordings, to make them more objective, behaviorally anchored, and developmentally sensitive to young children (Cohen & Scheeringa, 2009; Scheeringa, Zeanah, & Cohen, 2011). This approach used DSM-IV criteria as a starting point, but modified the criteria and studied the effects of those modifications in a series of studies on young children (Scheeringa, Peebles, Cook, & Zeanah, 2001; Scheeringa & Zeanah, 2008).

The major change is a modification to lower the requirement for the C criterion (numbing and avoidance items) from three out of seven items to just one out of seven items. While this modification was empirically driven and replicated in multiple studies, the rationale behind it was that the C criterion items reflect highly internalized phenomena that appear to be either developmentally impossible in early childhood or extremely difficult to detect even when present (Sheeringa, 2009). Particularly, a note for C4 item indicates that diminished interest in significant activities may be manifested as constricted play; Criterion C5 adds a note that feelings of detachment or estrangement “may be manifest in young children as social withdrawal.” This anchors the item in an observable behavior apparent to caregivers, with the implication that it is the behavioral manifestation of detachment and estrangement in young children (Fivush, 1999). The most important difference is a change in the threshold of Cluster C: in fact, many of the Cluster C symptoms are highly internalized phenomena that seem to be either developmentally impractical (e.g., sense of a foreshortened future) or difficult to detect even if they are present (e.g., avoidance of thoughts or feelings related to the traumatic event and inability to recall an important aspect of the event). Two symptoms that were deemed inappropriate for the developmental capacities of young children (e.g., sense of foreshortened future and inability to recall aspects of the trauma) were also removed. Finally, the criterion thresholds were modified so that only one symptom was required for avoidance (instead of three) and hyperarousal (instead of two).

Other changes included omitting Criterion A2, as it is difficult to determine a young child's subjective experience of an event, especially if there are no witnesses to his or her reaction (Scheeringa et al., 1995). As a matter of fact, “fear, helplessness, or horror” at the time of a traumatic event are difficult to be determined for young children who cannot accurately report their own experience, especially if there are no adults present to witness their reactions. DSM-IV specifically notes that children may display “disorganized or agitated” behavior instead of “fear, helplessness, or horror”, but it is not clear what “disorganized” or even “agitated” behavior refers to, nor what data support their use as substitutes for “fear, helplessness, or horror”.

The B1 symptom is phrased as “recurrent and intrusive distressing recollections of the event, including images, thoughts, or perceptions”: all three conditions—recurrent, intrusive, and distressing—are necessary. The new PTSD algorithm indicates that preschool children do not always manifest distress, even when they report intrusive thoughts or at least seem preoccupied by traumatic reminders.

Finally, Criterion D2 requires “Irritability or outbursts of anger” but the PTSD-AA modifies this criterion with the addition of “extreme temper tantrums.” The behavior being described has to be new onset or worsening of prior tantrum intensity or frequency following a traumatic event for it to be counted as endorsed.

Following further validations of the PTSD-AA, the algorithm was refined to reflect findings proving that the hyperarousal threshold should be kept at two or more symptoms (Scheeringa et al., 2003; Table 3).

**Table 3:** DSM-IV-R criteria for posttraumatic stress disorder (PTSD) showing alternative algorithm changes (adapted by Scheeringa, Zeanah, & Cohen, 2011)

Diagnostic criteria for posttraumatic stress disorder
A. The person has been exposed to a traumatic event in which the following were present:
(1) the person experienced, witnessed, or was confronted with an event or events that involved actual or threatened death or serious injury, or a threat to the physical integrity of self or others (no change from DSM-IV).
B. The traumatic event is persistently re-experienced in one (or more) of the following ways:
(1) recurrent and intrusive distressing recollections of the event, including images, thoughts, or perceptions. Note: In young children, repetitive play or repetitive *behaviors* may occur in which themes or aspects of the trauma are expressed.
Furthermore, recollections may appear not to be distressing in young children
(2) recurrent distressing dreams of the event. Note: In children, there may be frightening dreams without recognizable content;
(3) acting or feeling as if the traumatic event was recurring (includes a sense of reliving the experience, illusions, hallucinations, and dissociative flashback episodes, including those that occur on awakening or when intoxicated). Note: In young children, trauma-specific reenactment may occur;
(4) intense psychological distress at exposure to internal or external cues that symbolize or resemble an aspect of the traumatic event;
(5) physiological reactivity on exposure to internal or external cues that symbolize or resemble an aspect of the traumatic event.
C. Persistence avoidance of stimuli associated with the trauma and numbing of responsiveness (not present before the trauma), as indicated by one or more of the following:
(1) efforts to avoid thoughts, feelings, or conversations associated with the trauma;
(2) efforts to avoid activities, places, or people that arouse recollections of the trauma;
(3) inability to recall an important aspect of the trauma;
(4) markedly diminished interest or participation in significant activities. Note: *In young children, this may be manifest as constriction in play;*
(5) feeling of detachment or estrangement from others (e.g., unable to have loving feelings). Note: *In young children, this may be manifest as social withdrawal;*
(6) restricted range of affect
(7) sense of foreshortened future (e.g., does not expect to have a career, marriage, children, or a normal life span).
D. Persistent symptoms of increased arousal (not present before the trauma), as indicated by two (or more) of the following:
(1) difficulty falling or staying asleep;
(2) irritability, outbursts of anger, or extreme temper tantrums in young children;
(3) difficulty concentrating;
(4) hypervigilance;
(5) exaggerated startle response.
E. Duration of the disturbance (symptoms in Criteria B, C, and D) is more than 1 month
F. The disturbance causes clinically significant distress or impairment in social, occupational, or other important areas of functioning

The validity of the PTSD-AA and DSM-IV algorithms have been examined to demonstrate convergent, discriminant, and predictive validity of each (Scheeringa, Zeanah, & Cohen, 2010, for a review). The data highlight a substantial confirmation of discriminant and predictive validity of the PTSD-AA algorithm with respect to the DSM-IV algorithm, to detect and diagnose the post-traumatic symptoms in early childhood.

The application of these modifications, aimed to find an alternative algorithm for PTSD, has produced broader opportunities to classify and diagnose the disorder: the rate of PTSD in a population that has not sought help after a gas explosion in Japan was 25% (Ohimi et al., 2002) and amounted to 26% when considering samples of non-clinical children exposed to a variety of traumatic events (Scheeringa et al., 2003), whereas rates of PTSD using the DSM-IV criteria in both studies were 0%. Similarly, the rate of the disorder amounted to over 40% in clinic-referred children who witness domestic violence (Ghosh Ippen, Briscoe-Smith, & Lieberman, 2004) and to 60–69% in samples of children exposed to a variety of traumas, while adopting DSM-IV criteria the percentage was 2% for the first research and 13–20% for the second. In summary, the rates of PTSD in infancy and early childhood are consistent with rates found in older populations when developmentally sensitive measures and criteria are used.

On the basis of these data, some authors suggest a few recommendations for the Diagnostic and Statistical Manual of Mental Disorders, Fifth Edition (DSM-V; American Psychiatric Association, 2013) to consider the developmental differences in the expressions of disorders in different age groups more completely than the earlier editions of DSM have done. The attempt to introduce a specifically developmental point of view and developmental considerations in the definition and classification of psychopathology in childhood is included in the text descriptions of each disorder. In addition, prior research suggests that individuals of different ages may express features of the same specific criteria somewhat differently (Scheeringa et al., 2011). The descriptions of criteria in DSM-V may need, also, to be modified to reflect age differences in manifestations of the same underlying criteria. Finally, there may be sufficient differences in the expressions of some disorders to justify an age-related subtype of the disorder.

In general, PTSD remains a well-validated disorder, and it is the most useful construct of child post-trauma for research and clinical developmental psychopathology, but the current PTSD diagnostic criteria should be revised to reflect current research about developmental manifestations of this disorder. In fact, the proposed criteria for the DSM-V contain substantial changes from DSM-IV, but no studies with preschool children have empirically analyzed these criteria. From initial inspection, the proposed criteria seem to recreate the same problems for young children of earlier iterations of PTSD criteria. The threshold problem of Cluster C for young children that had been noted for the DSM-IV criteria may have been exacerbated. Instead of requiring three symptoms from the DSM-IV Cluster C (out of a possible seven), this cluster has been split into two new clusters, namely C and D, and four symptoms are now required (out of a possible 10). Furthermore, several Cluster C symptoms are highly internalized and regard abstract phenomena limiting their applicability to preschool children. Even if young children experience these symptoms, their limited verbal capacities to express or self-report the symptoms make them difficult to detect. This problem has not been addressed in the proposed criteria, because the two new symptoms added to the new cluster D are both highly internalized (self-blame and a negative emotional state of fear, horror, anger, guilt, or shame) and require sophisticated verbal capacities to be expressed. Moreover, another symptom has been added to the increased arousal cluster (reckless or self-destructive behavior), namely the new cluster E, which recreates the same problem as many other DSM-IV symptoms, that is being difficult to detect reliably in children with absent or emerging verbal capacities. Increasing the threshold from two symptoms to three will most likely limit the applicability of these criteria to young children.

In light of these critical considerations of proposed criteria of PTSD in childhood for DSM-V classification, Scheeringa et al. (2011) suggest the following recommendations: (1) Criterion A2 should be dropped for preschool children. If retained, it should include a broader range of reactions including worry, sadness, crying, numbness, and confusion; (2) Criterion B1 should be broadened to include other emotional reactions during recollections other than distress; (3) criteria C4, C5, and D2 should be qualified slightly to illustrate their behavioral manifestations in preschool children; (4) the threshold for Criterion C symptoms should be dropped from 3 to 1.

## 3. Developmental trauma disorder

In an attempt to more clearly delineate a diagnosis for the symptomatic manifestations of PTSD in childhood, the Complex Trauma Taskforce of the National Child Traumatic Stress Network has fostered a new conceptualization of DTD to define the characteristics and the impact of interpersonal trauma exposure in childhood (van der Kolk, 2005a; van der Kolk & D'Andrea, 2010; Table 4). The definition “interpersonal trauma” refers to a range of maltreatment, interpersonal violence, abuse, assault, and neglect experiences, including familial physical, sexual, emotional abuse, and incest; community-, peer-, and school-based assault, molestation, and severe bullying; severe physical, medical, and emotional neglect; witnessing domestic violence; as well as serious and pervasive disruptions in caregiving as a consequence of severe caregiver mental illness, substance abuse, criminal involvement, or abrupt separation or traumatic loss (D'Andrea, Ford, Stolbach, Spinazzola, & van der Kolk, 2012). This composite definition of interpersonal trauma derives from definitions and categories utilized by the National Child Traumatic Stress Network (NCTSN; Pynoos, Steinberg, Layne, Briggs, Ostrowski, & Fairbank, 2009) as an adaptation of child trauma definitions established by the National Child Abuse and Neglect Data System (NCANDS; U.S. Department of Health and Human Services, 2011).

**Table 4:** Criteria for developmental trauma disorder (DTD; adapted by van der Kolk, 2005a)

A. Exposure
(a) Multiple or chronic exposure to one or more forms of developmentally adverse interpersonal trauma (e.g. abandonment, betrayal, physical assaults, sexual assaults, threats to bodily integrity, coercive practices, emotional abuse, witnessing violence and death)
(b) Subjective experience (e.g. rage, betrayal, fear, resignation, defeat, shame)
B. Triggered pattern of repeated dysregulation in response to trauma cues
Dysregulation (high or low) in the presence of cues. Changes persist and do not return to baseline; not reduced in intensity by conscious awareness.
Affective
Somatic (e.g. physiological, motoric, medical)
Behavioral (e.g. re-enactment, cutting)
Cognitive (e.g. thinking that is happening again, confusion, dissociation, depersonalization)
Relational (e.g. clinging, oppositional, distrustful, compliant)
Self-attribution (e.g. self-hate, blame)
C. Persistently altered attributions and expectancies
Negative self-attribution
Distrust of protective caretaker
Loss of expectancy of protection by others
Loss of trust in social agencies to protect
Lack of recourse to social justice/retribution
Inevitability of future victimization
D. Functional impairment
Educational
Familiar
Peer
Legal
Vocational

The aim is to consider two main aspects of the trauma in childhood: (1) the interpersonal nature of the trauma and (2) the sequence of developmental trauma. The construct, proposed by the authors, refers to the “complex trauma” concept, considering the interpersonal nature of the traumatic events that a person can experience during their development; the purpose is to introduce a broader description both of what can be considered a traumatic experience in childhood and of the symptomatology in reference to the experience.

In fact, whilst isolated traumatic events, such as medical trauma and accidents, probably produce discrete conditioned behavioral and biological responses to reminders of the trauma, chronic condition of exposure to several traumatic conditions has pervasive effects on the development of mind and brain. Surveys of childhood trauma (see van der Kolk & D'Andrea, 2010 for a review) reveal a relatively low prevalence of childhood exposure to non-interpersonal traumas such as accidents, disasters, or severe illness compared to exposure to intrafamilial traumas, such as physical abuse, emotional abuse, neglect and exposure to domestic violence.

Similar to the previously cited authors, the DSM IV has a PTSD diagnosis that does not capture the developmental impact of childhood trauma. The diagnosis of PTSD is not developmentally sensitive and does not adequately describe the effect of exposure to childhood trauma on the developing child, because infants and children who experience multiple forms of abuse often experience developmental delays across a broad spectrum, including the complex disruptions of affect regulation, the disturbed attachment patterns, the rapid behavioral regressions and shifts in emotional states, the loss of autonomous strivings, the aggressive behavior against self and others, the failure to achieve developmental competencies; the loss of bodily regulation in the areas of sleep, food and self-care; the altered schemas of the world; the anticipatory behavior and traumatic expectations; the multiple somatic problems, from gastrointestinal distress to headaches; the apparent lack of awareness of danger and resulting self endangering behaviors; the self-hatred and self-blame and the chronic feelings of ineffectiveness. These manifestations of chronic dysregulation may be triggered by genuine environmental threats, perceived threats resulting from the child's misinterpretation of actual events, and/or the child's extreme response to seemingly innocuous stimuli (van der Kolk, 2005b). In this view, for the absence of a developmental oriented diagnosis that accurately captures the multiple exposure to traumas in childhood and the pervasive nature of disturbances related to these traumas, children tend to receive a large amount of labels for any number of symptoms—PTSD and attention deficit, conduct, and mood disorders—that are treated as separate conditions. Approaching each of these problems one by one, rather than as expressions of a vast system of internal disorganization, runs the risk of losing sight of the forest in favor of one tree (van der Kolk, 2005a). In other words, an overly simplified diagnosis can lead to inadequate treatment and a poor outcome.

The new diagnosis called DTD is organized around the issue of triggered dysregulation in response to traumatic reminders, stimulus generalization, and the anticipatory organization of behavior to prevent the recurrence of the trauma impact. It is predicated that multiple exposures to interpersonal trauma have consistent and predictable consequences, which affect many areas of functioning. The definition of the DTD is an alternative way to introduce a new diagnosis in the DSM that accounts for the child complex exposure to the trauma more completely.

This diagnosis is based on the belief that multiple exposures to interpersonal trauma, such as abandonment, physical or sexual assaults or witnessing domestic violence have consistent and predictable consequences that affect many areas of functioning. These experiences bring about (1) intense affects such as rage, fear, resignation, defeat and shame and (2) efforts to ward off the recurrence of those emotions, including the avoidance of experiences that facilitate them or engaging in behaviors that convey a subjective sense of control in the face of potential threats. The children exposed to interpersonal trauma tend to behaviorally reenact their traumas either as perpetrators, in aggressive or sexual acting out against other children, or in frozen avoidance reactions. They show patterns of physiological dysregulation that may lead to multiple somatic problems, such as headaches and stomach aches in response to fearful and helpless emotions. They anticipate and expect the trauma to reoccur and respond with hyperactivity, aggression, defeat, or freeze responses to minor stresses. All of these problems are expressed in dysfunction in multiple areas of child functioning (van der Kolk, 2005b) or in a possible subsequent symptomatology (i.e., adolescence), even if there are no empirical studies that have confirmed these constructs, particularly in childhood:

1. Attachment behaviors and bonds (interpersonal problems and poor affect attunement in relationships);
2. Biological functioning (somatic symptoms, analgesia, organic symptomatology in life course);
3. Emotion regulation (affective dysregulation, poor ability of identifying and discriminating emotions in self and others, difficulties in communicating one's own needs and desires);
4. dissociative state symptoms (impairment of the normal state of awareness, depersonalization, amnesia);
5. behavioral self-control (aggression directed toward the self and toward others, opposition to others, substance abuse, risk behaviors);
6. cognition (attention disorders, learning disabilities, information processing disorders, low planning ability);
7. self-identity (body dysmorphic disorder, low self-esteem, shame, sense of guilt).

In these authors’ opinion, no single current psychiatric diagnosis accounts for the cluster of symptoms that research has shown frequently to occur in children exposed to interpersonal trauma. The DSM-IV-R (American Psychiatric Association, 2000b) has only one diagnosis that specifically identifies trauma as an antecedent, PTSD, which does not capture the spectrum of post-trauma symptoms among children. Differently, studies on the sequence of serial or repeated childhood maltreatment, neglect, and interpersonal violence demonstrate that these experiences place children at risk of chronic and severe coexisting problems with emotion regulation, impulse control, attention and cognition, dissociation, interpersonal relationships and attributions (D'Andrea, Ford, Stolbach, Spinazzola, & van der Kolk, 2012). These data highlight the co-occurrence of multifaceted symptoms in children exposed to several traumatic experiences, that range across the spectrum of disorders (Finkelhor, Ormrod, & Turner, 2007). This available evidence suggests that the sequence of exposure to childhood interpersonal trauma may constitute the basis for a distinct new psychiatric diagnosis or, perhaps, a construct or framework within which to research this topic. Therefore, further research is needed to systematically develop and test the validity and clinical utility of a new diagnosis. In addition, a diagnosis based upon exposure to developmentally adverse interpersonal trauma in childhood has the potential to alert clinicians to the influential role of childhood trauma in psychopathology (Ford, 2005; van der Kolk, 2005a).

In summary, the concept of DTD is based on these following criteria: (1) childhood exposition to an interpersonal trauma is followed by a spectrum of specific symptoms; (2) the interpersonal nature of the trauma allows some experiences to be considered as both the cause of trauma for a child and as symptomatological expression of the trauma itself. For example, a disorganized attachment relation with the parent may lead to a traumatic experience for the child or generate a dysfunctional attachment model, representing one of the clinical factors to be considered for the diagnosis; (3) these symptoms cannot be accounted for by any existing DSM-IV diagnosis or combination of comorbid diagnoses, including PTSD; (4) research on the biological systems disrupted by childhood trauma is consistent with this spectrum of behavioral, affective, cognitive, and relational symptoms; (5) the application of nonspecific diagnoses to children exposed to interpersonal trauma reduces the likelihood of positive treatment outcomes, whereas interventions that comprehensively address the spectrum of problems increase the likelihood of positive treatment outcomes.

## 4. Considerations on PTSD in infancy: the first year of life

In his review on this theme, Sheeringa (2009) reports that there have been no cases of infants (under 12 months) published in the literature that clearly meet criteria for PTSD: it still appears true that infants can develop PTSD but it is not clearly convincing if children aged <9–12 months can manifest the critical items of the PTSD symptomatology. In the author's view, below 9 months of age, infants can show distress from painful circumstances but it is not clear if there are the cognitive meta-associations of connection between various stimuli and threat reminders. In effect, symptoms in this age may be more akin to conditioning, which disappears relatively quickly with the removal of the painful stimulus.

The developmental problems in the definition and classification of PTSD in the first year of life suggest the need for a specific focus on the development and functioning of memory in early infancy; in fact, memory is a critical issue in PTSD. To demonstrate key signs of the disorder (distress and reminders of the event, intrusive recollections of the event, avoidance of the event, and/or flashback of the event) the person must have some form of memory of past events. The memory of past events is studied in its two components, relative to the age and the developmental level of the infant. For the implicit memory (behavioral, non-declarative memory), tests have attested that at age of 6 months, infants show behavior that demonstrate their capacity to reenact traumatic experiences (Collie & Hayne, 1999). These results are in line with the research by Nelson (1995) that hypothesizes that the brain structure for long-term memory is in place and functional by at least 8–9 months of life. In other words, in the early childhood there are the neuro-biological, cognitive, and behavioral competencies that support the behavioral memories, such as automatic distress reactions to situations that resemble the experience of individual's past events. However, the autobiographical memory (explicit and declarative memory) appears approximately at 18 months of age, in line with the emergence of language, increases between 18 and 36 months and manifests in a coherent narrative form after 36 months. It is a kind of memory that permits the expression of personal experiences in the form of verbal recalls. For this reason, memory research suggests that infants who are traumatized at the age of 6 months or earlier will not have retrievable autobiographical narrative of these experiences. In general, research on this theme concluded that children under 24 months of age failed to provide a coherent narrative of the traumatic event (Peterson & Whalem, 2001) even if they clearly recall stressful experiences, and these recollections are at least as detailed as normative events (Fivush, 1999). Nevertheless, in early childhood it is possible that the infants show only behavioral evidence of recalling previous experiences but are not able to express memories of these events in verbal narrative form.

## 5. Conclusions and perspectives

Defining and applying specific criteria for PTSD diagnosis in childhood, especially during the pre-school years, represent nowadays two of the main purposes in research and clinical studies on childhood trauma. This was the aim that animated all the efforts directed to modify criteria for the fifth edition of DSM, to make them developmentally sensitive; nevertheless, they are still not adequate in some authors’ opinion. In fact, although no final conclusions can be made, the proposed changes in PTSD criteria do not seem likely to be applicable across the lifespan without modification.

Future research is needed to examine the development and validation of developmentally appropriate assessment measures to clearly establish the prevalence comorbidity, and course of psychological morbidity in young traumatized children and their families (De Young, Kenardy, & Cobham, 2011). In summary, the establishment of empirically validated, developmentally sensitive PTSD diagnostic criteria for toddlers and preschoolers is one of the key tasks remaining for the DSM classification system. This is important in order to increase awareness of preschool mental health and to facilitate the development of gold standard assessment instruments and promote the development and evaluation of evidence-based treatments.

However, a complex trauma definition is consistent with a more specific definition of the concept of trauma and with a more nuanced understanding of the impact of different types of exposure to trauma (Reid-Quinones et al., 2011) and individual differences in exposure and reactions to trauma (Voisin, Neilands, & Hunnicutt, 2011), particularly in childhood. The authors consider that a diagnosis describing complex trauma may help not to define complex trauma subjects as pathological; in effect, they're often labeled with many diagnoses that can become a source of chronic stigma. However, the goal is to advance research and clinical work, rather than the “reification of diagnosis” (Hyman, 2010).

This aim is a fundamental agenda that will require a specific series of studies over many years, to understand the clinical and social needs of trauma exposed children (and their families, communities, and society) and prevent subsequent psychopathology in their life cycle.

## References

[CIT0001] American Psychiatric Association (1994). Publication manual of the American Psychological Association.

[CIT0002] American Psychiatric Association (2000b). Diagnostic and statistical manual of mental disorders.

[CIT0003] American Psychiatric Association (2013). Diagnostic and statistical manual of mental disorders.

[CIT0004] Anthony J. L, Lonigan C. J, Hecht S. A (1999). Dimensionality of posttraumatic stress disorder symptoms in children exposed to disaster: Results from confirmatory factor analyses. Journal of Abnormal Psychology.

[CIT0005] Carrion V. G, Weems C. F, Ray R, Reiss A. L (2002). Toward an empirical definitions of pediatric PTSD: The phenomenology of PTSD symptoms in youth. Journal of the American Academy of Child & Adolescent Psychiatry.

[CIT0006] Child Maltreatment (2011). US Department of Health and Human Services. Administration on Children, Youth and Families.

[CIT0007] Cohen J, Scheeringa M (2009). Post traumatic stress disorders diagnosis in children: Challenges and promises. Dialogues in Clinical Neuroscience.

[CIT0008] Collie R, Hayne H (1999). Deferred imitation by 6- and 9- month-old infants: More evidence for declarative memory. Developmental Psychobiology.

[CIT0009] D'Andrea W, Ford J, Stolbach B, Spinazzola J, van del Kolk B. A (2012). Understanding interpersonal trauma in children: why we need a developmentally appropriate trauma dyagnosis. American Journal of Orthopsychiatry.

[CIT0010] Davidson J, Fairbank J, Daviddson J, Foa E (1993). The epidemiology of posttraumatic stress disorders. Post-traumatic stress disorders: DSM-IV and beyond.

[CIT0011] De Young A. C, Kenardy J. A, Cobham V. E (2011). Diagnosis of post traumatic stress disorders in preschool children. Journal of Clinical Child and Adolescent Psychology.

[CIT0012] Egger H. L, Erkanli A, Keeler G, Potts E, Walter B. K, Angold A (2006). Test–retest reliability for the Preschool Age Psychiatric Assessment (PAPA). Journal of the American Academy of Child & Adolescent Psychiatry.

[CIT0013] Finkelhor D, Ormrod R. K, Turner H. A (2007). Revictimization patterns in a national longitudinal sample of children and youth. Child Abuse & Neglect.

[CIT0014] Fivush R (1999). Children's recollections of traumatic and non traumatic events. Development and Psychopathology.

[CIT0015] Fletcher K. E, Masch E. J, Barkley R (1996). Childhood posttraumatic stress disorder. Child psychopathology.

[CIT0016] Ford J. D (2005). Treatment implications of altered neurobiology, affect regulation and information processing following child maltreatment. Psychiatric Annals.

[CIT0017] Ghosh Ippen C. G, Briscoe-Smith A, Lieberman A. F (2004). PTSD symptomatology in young children.

[CIT0018] Hyman S. E (2010). The diagnosis of mental disorders: The problem of reification. Annual Review of Clinical Psychology.

[CIT0019] Iselin G, Le Brocque R, Kenardy J, Anderson V, McKinlay L (2010). Which method of posttraumatic stress disorder classification best predicts psychosocial function in children with traumatic brain injury?. Journal of Anxiety Disorders.

[CIT0020] Kessler R, Sonnega A, Bromet E, Hughes M, Nelson C (1995). Posttraumatic stress disorder in the National Comorbidity Survey. Archives of General Psychiatry.

[CIT0021] Koenen K. C, Roberts A. L, Stone D. M, Dunn E. C, Lanius R. A, Vermetten E, Pain C (2010). The epidemiology of early childhood trauma. The impact of early life trauma on health and disease: The hidden epidemic.

[CIT0022] Laor N, Wolmer L, Mayes L, Gershon A (1997). Israeli preschool children under Scuds: A 30-month follow-up. Journal of the American Academy of Child & Adolescent Psychiatry.

[CIT0023] McFarlane A (1987). Posttraumatic phenomena in a longitudinal study of children following a natural disaster. Journal of the American Academy of Child & Adolescent Psychiatry.

[CIT0024] McNally R. J, Davidson J. R. T, Foa E. B (1993). Stressors that produce posttraumatic stress disorders in children. Posttraumatic stress disorders: DSM-IV and beyond.

[CIT0025] Meiser-Stedman R, Smith P, Glucksman E, Yule W, Dalgleish T (2008). The posttraumatic stress disorder diagnosis in preschool and elementary school-age children exposed to motor vehicle accidents. The American Journal of Psychiatry.

[CIT0026] Nelson C (1995). The ontogeny of human memory: A cognitive neuroscience perspective. Developmental Psychology.

[CIT0027] Ohimi H, Kojima S, Awai Y, Kamata S, Sasaki K, Tanaka Y (2002). Post-traumatic stress disorder in pre-school aged children after a gas explosion. European Journal of Pediatrics.

[CIT0028] Peterson C, Whalem N (2001). Five years later: Children's memories of medical emergencies. Applied Cognitive Psychology.

[CIT0029] Pynoos R. S, Steinberg A. M, Layne C. M, Briggs E. C, Ostrowski S. A, Fairbank J. A (2009). DSM–V PTSD diagnostic criteria for children and adolescents: A developmental perspective and recommendations. Journal of Traumatic Stress.

[CIT0030] Reid-Quinones K, Kliewer W, Shields B. J, Goodman K, Ray M. H, Wheat E (2011). Cognitive, effective, and behavioral responses to witnessed versus experienced violence. American Journal of Orthopsychiatry.

[CIT0031] Salmon K, Bryant R. A (2002). Post traumatic stress disorders in children: The influence of developmental factors. Clinical Psychology Review.

[CIT0032] Scheeringa M. S, Haslett N (2010). The reliability and criterion validity of Diagnostic Infant and Preschool Assessment (DIPA): A new diagnostic instrument for young children. Child Psychiatry and Human Development.

[CIT0033] Scheeringa M. S, Peebles C. D, Cook C. A, Zeanah C. H (2001). Toward establishing procedural, criterion, and discriminant validity for PTSD in early childhood. Journal of the American Academy of Child & Adolescent Psychiatry.

[CIT0034] Scheeringa M. S, Zeanah C. H (2008). Reconsideration of harm's: onsets and comorbidity patterns in preschool children and their caregivers following Hurricane Katrina. Journal of Clinical Child and Adolescent Psychology.

[CIT0035] Scheeringa M. S, Zeanah C. H, Cohen J. A (2010). PTSD in children and adolescents: toward an empirically based algorithm. Depression and Anxiety. Advance online publication.

[CIT0036] Scheeringa M. S, Zeanah C. H, Cohen J. A (2011). PTSD in children and adolescents: towards an empirically based algorithm. Depression and Anxiety.

[CIT0037] Scheeringa M. S, Zeanah C. H, Drell M. J, Larrieu J. A (1995). Two approaches to the diagnosis of posttraumatic stress disorders infancy and early childhood. Journal of the American Academy of Child & Adolescent Psychiatry.

[CIT0038] Scheeringa M. S, Zeanah C. H, Myers L, Putnam F. W (2003). New finding on alternative criteria for PTSD in preschool children. Journal of the American Child and Adolescent Psychiatry.

[CIT0039] Scheeringa M. S, Zeanah C. H, Myers L, Putman F (2005). Predictive validity in a prospective follow-up of PTSD in a preschool children. Journal of the American Academy of Child & Adolescent Psychiatry.

[CIT0040] Shaw M. D (1996). Twenty-one month follow-up study of school-age children exposed to hurricane. Journal of the American Academy of Child & Adolescent Psychiatry.

[CIT0041] Sheeringa M. S, Zeanah C. H (2009). Posttraumatic stress disorder. Handbook of Infant Mental Health.

[CIT0042] Taylor S, Kuch K, Koch W. J, Crockett D. J, Passey G (1998). The structure of posttraumatic stress symptoms. Journal of Abnomal Psychology.

[CIT0043] Van der Kolk B, D'Andrea A, Lanius R. A, Vermetten E, Pain C (2010). Towards a developmental trauma disorder diagnosis for childhood interpersonal trauma. The impact of early life trauma on health and disease: The hidden epidemic.

[CIT0044] Van der Kolk B. A (2005a). Developmental trauma disorder: Toward a rational diagnosis for children with complex trauma histories. Psychiatric Annals.

[CIT0045] Van der Kolk B. A (2005b). Child abuse and victimization. Psychiatric Annals.

[CIT0046] Voisin D. R, Neilands T. B, Hunnicutt S (2011). Mechanisms linking violence exposure and school engagement among African American adolescents: Examining the roles of psychological problem behaviors and gender. American Journal of Orthopsychiatry.

[CIT0047] Zero to Three (2005). Diagnostic classification 0–3R: Diagnostic classification of mental health and developmental disorders in infancy and early childhood.

